# Validation of the Intermountain patient perception of quality (PPQ) survey among survivors of an intensive care unit admission: a retrospective validation study

**DOI:** 10.1186/s12913-015-0828-x

**Published:** 2015-04-14

**Authors:** Samuel M Brown, Glen McBride, Dave S Collingridge, Jorie M Butler, Kathryn G Kuttler, Eliotte L Hirshberg, Jason P Jones, Ramona O Hopkins, Daniel Talmor, James Orme, for the Center for Humanizing Critical Care

**Affiliations:** Pulmonary and Critical Care Medicine, Intermountain Medical Center, 5121 S Cottonwood St, Murray, UT USA; Pulmonary and Critical Care Medicine, University of Utah School of Medicine, 26 North 1900 East, Salt Lake City, UT USA; Center for Humanizing Critical Care, Intermountain Healthcare, 5121 S Cottonwood St, Murray, UT USA; Strategic Planning and Research, Intermountain Healthcare, 36 S. State St., Salt Lake City, UT USA; Office of Research, Intermountain Healthcare, 5121 S Cottonwood St, Murray, UT USA; Geriatrics Research Education and Clinical Center (GRECC), Veterans Affairs Medical Center, Salt Lake City, UT USA; Department of Internal Medicine, Geriatrics Division, University of Utah School of Medicine, 30 N 1900 E, Salt Lake City, UT USA; Homer Warner Center for Informatics Research, Intermountain Healthcare, 5171 South Cottonwood Street, Suite 220, Murray, UT USA; Pediatric Critical Care, University of Utah, 26 North 1900 East, Salt Lake City, UT USA; Kaiser-Permanente Southern California, 100 S Los Robles Ave, Pasadena, CA USA; Psychology Department and Neuroscience Center, Brigham Young University, 1022 SWKT, Provo, UT USA; Anesthesia, Critical Care and Pain Medicine, Beth Israel Deaconess Medical Center and Harvard Medical School, 1 Deaconess Rd, Boston, MA USA; Shock Trauma ICU, Intermountain Medical Center, 5121 S. Cottonwood Street, Murray, UT 84107 USA

**Keywords:** Intensive care, Patient satisfaction, Healthcare quality, Patient experience

## Abstract

**Background:**

Patients’ perceptions of the quality of their hospitalization have become important to the American healthcare system. Standard surveys of perceived quality of healthcare do not focus on the Intensive Care Unit (ICU) portion of the stay. Our objective was to evaluate the construct validity and internal consistency of the Intermountain Patient Perception of Quality (PPQ) survey among patients discharged from the ICU.

**Methods:**

We analyzed prospectively collected results from the ICU PPQ survey of all inpatients at Intermountain Medical Center whose hospitalization included an ICU stay. We employed principal components analysis to determine the constructs present in the PPQ survey, and Cronbach’s alpha to evaluate the internal consistency (reliability) of the items representing each construct.

**Results:**

We identified 5,680 patients who had completed the PPQ survey. There were three basic domains measured: nursing care, physician care, and overall perception of quality. Most of the variability was explained with the first two principal components. Constructs did not vary by type of respondent.

**Conclusions:**

The Intermountain ICU PPQ survey demonstrated excellent construct validity across three distinct constructs. This, in addition to its previously established content validity, suggests the utility of the PPQ survey as an assay of the perceived quality of the ICU experience.

**Electronic supplementary material:**

The online version of this article (doi:10.1186/s12913-015-0828-x) contains supplementary material, which is available to authorized users.

## Background

Both the technical quality and consumer perception of the quality of healthcare have become pressing issues in the contemporary American medical system. The Hospital Consumer Assessment of Healthcare Providers and Systems (HCAHPS) survey [1-3] is the best-known survey that is used to measure and improve patient-relevant quality outcomes. However, the HCAHPS is not specific to the ICU portion of a hospitalization, which may limit its applicability to improving the quality of care within the ICU. Intermountain Healthcare, a large, non-profit network of hospitals and clinics in the Intermountain West, has been measuring quality and patient-perceived quality for two decades. As part of this effort, in the 1990s Intermountain developed the Patient Perception of Quality (PPQ) survey through an iterative process intended to develop a “taxonomy of inpatient experiences.” Using long- and short-form structured interviews with hospital personnel (primarily physicians and nurses), hospital administrators, and recently discharged patients (300 randomly selected patients recently discharged from any of 10 Intermountain hospitals), constructs contained within the resulting PPQ survey were inductively defined from qualitative analysis. Themes within these structured interviews included attention to processes of care and identified multiple healthcare workers whose influence may have been important to patient experience. Survey items were developed from constructs identified in the initial phase and were then pilot tested in another 300 patients who had received inpatient care within the following departments of Intermountain hospitals: labor and delivery, orthopedics, neurology, medical-surgical, rehabilitation, cardiothoracic surgery, and ICU [4-6]. Intermountain subsequently administered the resulting PPQ to patients admitted to an ICU, asking them (or a family member) to comment specifically on their experience with the ICU as distinct from their experience with the hospital admission overall.

In order to better understand the characteristics of the PPQ ICU survey, we undertook a principal components analysis of the PPQ responses completed by patients, or their surrogates, admitted to an ICU during an index hospitalization over a five-year period.

## Methods

The PPQ is a 26-item, approximately 635-word survey that queries the “caring and concern” demonstrated by multiple types of healthcare workers as well as how well the healthcare workers “listened and seriously considered” what the patient communicated. Other topics include privacy, respect, clinical skill, ability to explain information, and shared decision making. The entire survey instrument is included in Additional file 1.

We analyzed results of the PPQ ICU survey administered to inpatients or their surrogates discharged from Intermountain Medical Center (IMC) from 2008–2012, inclusive. IMC is a 454-bed academic tertiary referral hospital in Salt Lake City, Utah with 84 ICU beds distributed across five adult ICUs. The Intermountain ICU PPQ survey was administered entirely independently of and subsequent to the HCAHPS survey and asked respondents to answer with regard to their ICU experience rather than in regards to their overall hospitalization. The PPQ ICU survey (see Table 1 and the Additional file 1) was administered exclusively by telephone. During the scripted survey encounter, a single respondent was identified from among patient, spouse, parent, other family member, or friend. Respondents other than the patient were interviewed only when the patient poorly remembered the ICU stay or was not able to respond to the survey at the time of telephone contact. Up to five telephone attempts were made for each survey, after which the potential respondent was classified as unreachable. While monthly reports of survey disposition (e.g., unable to contact, refused participation, etc.) were reported, survey-level disposition data is not maintained on the PPQ ICU survey, and, owing to a change in telephone survey vendors, the disposition reports are no longer available.

**Table 1:** **PPQ Items clustered by posited group, with distribution**

**Variable**	**Item**	**Percent with top score**	**Mean (SD)**
	**Physician**		
PHCC	Physician caring and concern	61	4.42 (0.88)
PHSK	Physician skill	69	4.57 (0.75)
PHEX	Did the physician explain?	59	4.35 (0.97)
	**Nurse**		
NUCC	Nurse caring and concern	67	4.53 (0.79)
NUSK	Nurse skill	63	4.50 (0.76)
NUFL	Did the nurse followup?	56	4.34 (0.91)
NUEX	Did the nurse explain?	56	4.35 (0.91)
NUCO	Nurse listening/consideration of your insights	58	4.37 (0.91)
	**Pain Control**		
CLPN	How well was your pain controlled?	56	4.31 (0.94)
	**Housekeeping**		
HKRM	Was your room clean?	60	4.44 (0.81)
	**Teamwork and Privacy**		
STPV	Did staff respect your privacy?	63	4.49 (0.78)
STTM	Did the teamwork together to coordinate care?	57	4.38 (0.85)
TRIN	Did the team prepare you to leave the ICU?	51	4.21 (1.01)
STDE	Team incorporated your concerns into decision making	51	4.21 (1.01)
	**General quality**		
OVCS	Overall quality of care provided	61	4.45 (0.82)
CLBE	Confidence the ICU provided best care possible	72	4.62 (0.74)

All items are on a 5-point Likert scale.

For validation we performed a principal components analysis (PCA) of the PPQ ICU survey results and then calculated item-total correlations and Cronbach’s Alpha for identified factors. PCA is a mathematical technique for simplifying a large number of variables by identifying patterns of covariance among them. These covariance patterns can be expressed as a few new variables that are weighted combinations of the many original variables (the weights are often called “loadings” by convention). These new variables are called the “principal components” of the data and represent important underlying structure in the data. PCA thereby allows empirical determination of what constructs (components) the PPQ measures, an additional level of validation important to establishing the validity of the PPQ. PCA is also important because it identifies which questions can be aggregated so that component themes can be compared with tests of statistical significance in future research. In addition, we evaluated the reliability of the questions loading onto each component with Cronbach’s Alpha test of internal consistency. Items most closely associated with a given component are likely to reflect the same underlying construct; the Cronbach’s Alpha measures the correlation among items belonging to the same construct.

We excluded patients who were on the Intermountain “do not call” list and patients admitted to an ICU under “observation” status, such as for brief monitoring after an invasive procedure. We included only respondents who could complete the survey in English; no non-English survey materials were available.

The Intermountain Healthcare Institutional Review Board exempted this quality improvement project from the requirement for informed consent. The Intermountain Privacy board approved publication of these results.

### Statistical methods

We report central tendencies as mean (normally distributed data) or median (non-normally distributed data). We compared between or among group central tendencies with Fisher’s exact, Student’s *t*-test, multiple ANOVA, Wilcoxon rank-sum, or Kruskal-Wallis statistic as dictated by type of comparison and normality of the data.

For factor/construct analysis we employed principal components analysis with oblique rotation to allow for correlation among the factors. Specifically we compared the factors identified on these analyses to the constructs proposed during the development of the ICU PPQ (“physician quality”, “nurse quality”, “pain control”, “housekeeping”, “teamwork and privacy”, “general quality”). After the factors were extracted and identified, we evaluated the internal consistency of each with Cronbach’s Alpha test of reliability. We performed a sensitivity analysis that evaluated the stability of the construct/factor analysis for different respondents. Our primary approach to missing data was to restrict our analysis to complete cases (listwise deletion). In a sensitivity analysis, we imputed the mean value of the item for missing items.

We performed all analyses in SPSS and the R Statistical Package, version 3.01 [7].

## Results and Discussion

**Figure 1:**
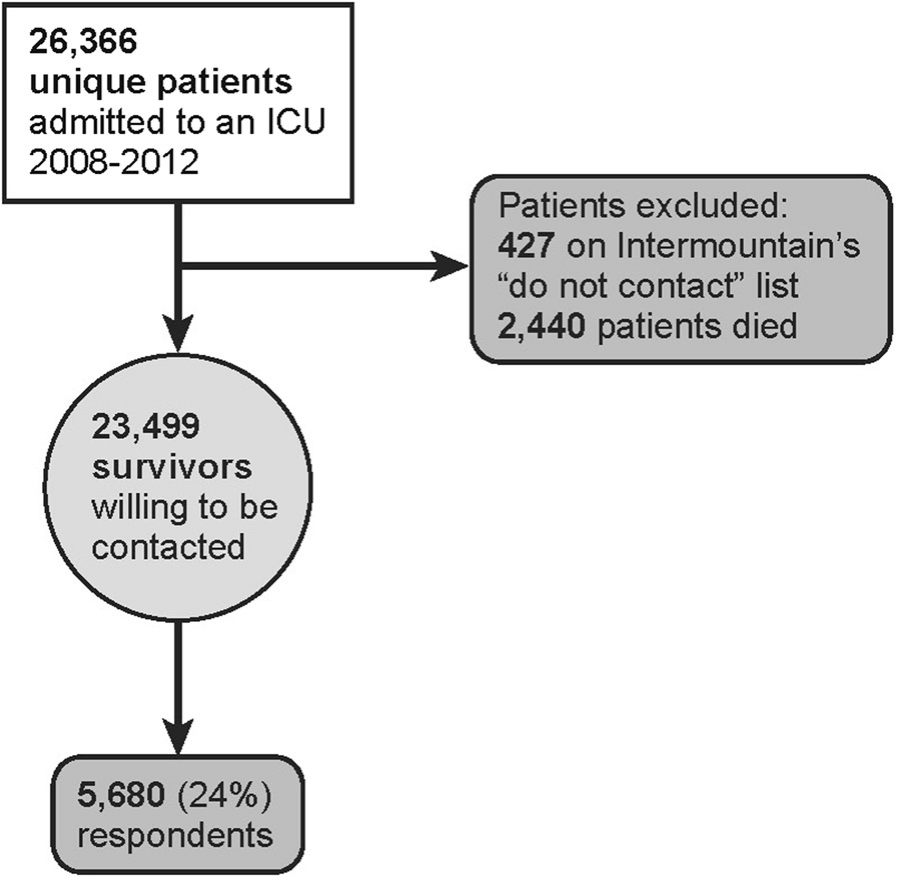
Flow-chart representing patient selection process by which survey respondents were identified.

From 2008–2012, inclusive, 26,366 unique inpatients were admitted to an IMC ICU, of whom 2,440 died before a survey could be completed. Figure 1 summarizes the strategy that identified 5,680 inpatient admissions associated with a completed PPQ ICU survey. Twenty-four percent of eligible respondents completed a survey. Missing data occurred in 0-8% of individual items on the survey: 4,087 (72%) surveys represented complete surveys for the 16 items of interest. Respondents included primarily spouses (N = 2,208; 39%), parents (N = 1,642; 29%), and patients (N = 1,411; 25%), with the rest classified as “other.” Table 1 displays the PPQ items, grouped by posited underlying construct, as well as the mean and standard deviation for those items. All items were negatively skewed, with median of 5 (inter-quartile range of 4–5) out of 5 possible points. The proportion of respondents giving the “top score” (“Excellent” or “Always”) in each category ranged from 51% to 72%, as displayed in Table 1.

**Figure 2:**
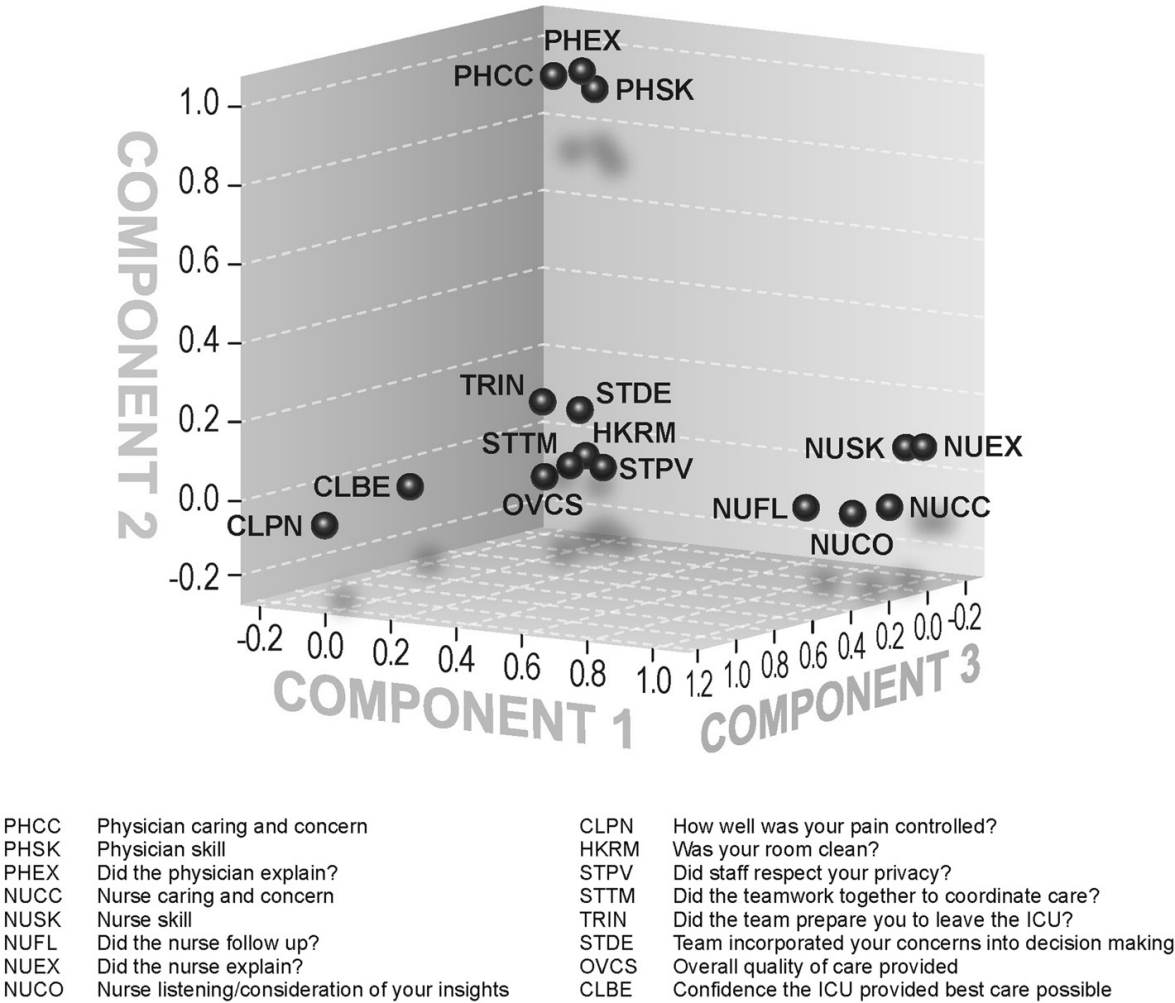
Plot of principal components of individual items. The components are the axes; individual items are depicted by their locations within the three major axes of the principal components analysis.

In principal components analysis (results depicted in Table 2), the first component (58.50% of variance) referred to nursing elements with loadings of 0.77-0.97 in the pattern matrix for all five nurse-related items. The second component (7.73% of variance) clearly distinguished three physician-related items from all other items with loadings of 0.91-0.95. The third component (4.57% of variance) was represented by six items related to overall quality of care in the hospital with loadings of 0.60-1.0. The items on cleanliness and privacy loaded onto none of the three components. Taken together, the three components that we identified accounted for 71% of the total variance. Figure 2 displays a component plot of the first three components, visually demonstrating the dimension reduction effected by PCA. Diagnostics for the PCA suggested adequate decomposition: (A) Determinant value of 0.0000065 was less than 0.00001 indicating that dimension reduction is indicated, (B) Overall Kaiser–Meyer–Olkin (KMO) value of 0.964 was superb indicating that correlation patterns were compact enough to elicit reliable and distinct factors, and (C) Bartlett’s Test of Sphericity was significant (p < 0.001) indicating that the population correlation matrix for our items was significantly different from the identity matrix. Cronbach’s Alpha for the items within nursing, physician, and overall care components were 0.92, 0.89, and 0.90 respectively, suggesting excellent inter-item correlation within each component. The overall Cronbach’s Alpha for all items was 0.95, although the correlation matrix (Table 3) suggested no evidence of redundancy within the correlation matrix.

**Table 2:** **Pattern matrix of principal components analysis**

	**Component**			
	**1**	**2**	**3**	**4**
**Nursing items**				
Did the nurse explain?	**.971**	.077	-.123	-.074
Nurse caring and concern	**.967**	-.056	.024	-.110
Nurse skill	**.916**	.066	-.150	.027
Nurse listening/consideration of your insights	**.877**	-.073	.065	-.020
Did the nurse follow up?	**.767**	-.061	.119	.038
**Physician items**				
Did the physician explain?	-.011	**.947**	-.041	-.019
Physician caring and concern	-.040	**.944**	.040	-.052
Physician skill	.028	**.905**	-.043	-.001
**Overall items**				
How well was your pain controlled?	-.148	-.071	**1.044**	-.085
Confidence the ICU provided best care possible	.046	.012	**.925**	-.195
Overall quality of care provided	.269	.050	**.596**	.002
**Independent items**				
Did the team work together to coordinate care?	.254	.044	.452	.189
Team incorporated your concerns into decision making	.238	.162	.381	.167
Did the team prepare you to leave the ICU?	.116	.165	.375	.240
Was your room clean?	-.081	-.046	-.162	1.114
Did staff respect your privacy?	.231	.004	.269	.383

Extraction method: Principal component analysis; Rotation method: Promax with Kaiser Normalization. The loadings most prominent in a given principal component are bolded.

**Table 3:** **Correlation Matrix for 11 Questions loading onto at least one component**

		**phcc**	**phsk**	**phex**	**nucc**	**nusk**	**nufl**	**nuex**	**nuco**	**clpn**	**clbe**	**ovcs**
phcc	Pearson	1	.754	.762	.450	.463	.473	.477	.451	.470	.483	.552
	N	5294	5190	5140	5284	5229	5180	5201	5197	4911	5263	5288
phsk	Pearson	.754	1	.701	.453	.492	.444	.472	.452	.455	.493	.542
	N	5190	5273	5128	5262	5217	5165	5175	5172	4897	5248	5268
phex	Pearson	.762	.701	1	.422	.438	.440	.502	.447	.444	.468	.521
	N	5140	5128	5221	5211	5156	5111	5137	5130	4842	5192	5215
nucc	Pearson	.450	.453	.422	1	.698	.705	.706	.732	.525	.593	.695
	N	5284	5262	5211	5666	5585	5514	5550	5538	5236	5613	5657
nusk	Pearson	.463	.492	.438	.698	1	.648	.699	.675	.509	.556	.633
	N	5229	5217	5156	5585	5597	5457	5496	5476	5178	5549	5588
nufl	Pearson	.473	.444	.440	.705	.648	1	.666	.737	.545	.579	.670
	N	5180	5165	5111	5514	5457	5524	5437	5446	5127	5490	5516
nuex	Pearson	.477	.472	.502	.706	.699	.666	1	.713	.508	.554	.647
	N	5201	5175	5137	5550	5496	5437	5562	5471	5146	5512	5553
nuco	Pearson	.451	.452	.447	.732	.675	.737	.713	1	.543	.581	.673
	N	5197	5172	5130	5538	5476	5446	5471	5547	5137	5511	5538
clpn	Pearson	.470	.455	.444	.525	.509	.545	.508	.543	1	.570	.596
	N	4911	4897	4842	5236	5178	5127	5146	5137	5247	5205	5242
clbe	Pearson	.483	.493	.468	.593	.556	.579	.554	.581	.570	1	.698
	N	5263	5248	5192	5613	5549	5490	5512	5511	5205	5626	5616
ovcs	Pearson	.552	.542	.521	.695	.633	.670	.647	.673	.596	.698	1
	N	5288	5268	5215	5657	5588	5516	5553	5538	5242	5616	5667

All correlations significant at p < 0.001 level (2-tailed).

On sensitivity analysis of the relationship between the PCA constructs and the identity (e.g. patient, spouse, parent, other family member, or friend) of the respondent, there was very little difference among the respondents beyond the “other” category, which had too few respondents (N = 46) to support a robust PCA. On the sensitivity analysis in which we imputed missing items, there was no substantial difference within the PCA. While differences in the overall hospital rating by respondent achieved statistical significance (p < 0.001 by Kruskal Wallis) the difference was relatively minor, with means varying from 4.33 (“other” respondent) on the low end to 4.54 (patient respondent) on the high end (median for all types of respondents was 5). The respondents did not differ in their overall physician rating (p = 0.64), while their assessment of nursing skill was significant (p = 0.002), with minor difference in the mean responses (“other” respondents’ responses were slightly lower, and patients’ responses were slightly higher than other types of respondents).

Notably, the overall hospital rating varied both by the specific ICU (p < 0.001 by Kruskal-Wallis) and by year of assessment (p < 0.001 by Kruskal-Wallis). Whereas the mean for overall hospital rating was 4.37 for 2008, it was 4.55 for 2012.

## Conclusions

In a large sample from all ICUs at a referral center in the Intermountain West, we found that the Intermountain ICU PPQ survey administered to ICU survivors and/or a member of their family primarily identified three constructs of perceived quality: overall quality of care, quality of nurses, and quality of physicians. The structure of the survey was similar across different classes of respondents. These data suggest that analyses of results from the ICU PPQ survey could be fruitfully summarized as composite scores on each of the three components and that the survey could be made more frugal through exclusion of items outside the three components. Overall, respondents were reasonably well satisfied with the quality of care they received. Our sample size (5680) compares favorably with the majority of studied instruments to measure perceived quality of care among hospitalized patients [8]. The ICU PPQ Survey is somewhat more frugal than HCAHPS overall, and the constructs apparent on our PCA are fewer than the 6 constructs apparent in the HCAHPS survey [9].

The ICU PPQ survey could serve as a useful complement to mandatory HCAHPS survey activity for the purposes of ICU quality improvement because the ICU PPQ survey is specific to the ICU experience and allows for non-patient respondents. Results of HCAHPS surveys will be affected by the hospital units (or emergency department) in which patients stayed before and after the ICU stay, making it more difficult to infer ICU quality performance from typical HCAHPS survey results. Other measures of ICU satisfaction have been studied, including the Family Satisfaction with ICU (FS-ICU) survey [10]. While specific to the ICU, the FS-ICU is restricted to family members only and is less frugal than the ICU PPQ Survey. Unfortunately, we were unable to make a direct comparison between the FS-ICU and the ICU PPQ survey in this study.

Overall, patients answered less frequently than spouses or parents and exhibited higher satisfaction than all other respondents. Whether the difference in perceived quality is because patients able to respond to the survey were healthier than patients unable to respond to the survey or because patients tend to rate healthcare experiences more favorably than their family members cannot be determined from the current study. We acknowledge that in other work, e.g., on quality of life, proxy and patient responses have correlated relatively poorly [11,12]. Unfortunately, we were unable to make a direct comparison of patient and proxy responses in the present study. The PPQ questionnaire appears to identify both temporal and inter-ICU differences, suggesting potential utility, although there remains a risk of bias related to patient populations (e.g., postoperative routine surgery versus major trauma) that may affect inter-ICU differences in scores.

We acknowledge that telephone surveys are consistently more positive than mail questionnaires in the HCAHPS survey [13]. The telephone mode may have contributed to overall higher scores on the PPQ, but this does not affect the construct validity presented in this study. Unfortunately, we do not have data on specific reasons for or distribution of non-response to this PPQ survey.

Per HCAHPS policy, we did not interview families of patients who died during or shortly after their hospital admission. We are therefore unable to comment on whether the PPQ survey could accurately capture satisfaction with ICU care during a hospitalization after which the patient did not survive.

In conclusion the Intermountain ICU PPQ survey demonstrated excellent construct validity across three distinct constructs: perceived quality of nurses, perceived quality of physicians, and overall perceived quality of the ICU. The construct validity of the ICU PPQ survey, in addition to its established content validity, suggests the utility of the ICU PPQ survey as an assay of the perceived quality of the ICU experience.

## Additional file

Additional file 1:
**The Intermountain PPQ Survey.**

## Abbreviations

FS-ICU: Family satisfaction in intensive care unit survey
HCAHPS: Hospital consumer assessment of healthcare providers and systems survey
ICU: Intensive care unit
IMC: Intermountain medical center
PCA: Principal components analysis
PPQ: Patient perception of quality survey
