# Supplementary material for: Validation of the Intermountain patient perception of quality (PPQ) survey among survivors of an intensive care unit admission: a retrospective validation study
Source: BMC Health Serv Res. 2015 Apr 14;15:155. doi: 10.1186/s12913-015-0828-x (PMC4429340; doi:10.1186/s12913-015-0828-x)
Supplement: Additional file 1: — The Intermountain PPQ Survey. [file 12913_2015_828_MOESM1_ESM.doc]

**Patient Perceptions of Quality**

**ICU Family/Patient Questionnaire**

***ADULT PATIENT INTRODUCTION***

Hello, this is ________ calling on behalf of (hospital). May I speak with (patient)?

*If asked for purpose of the call:* “We are calling to conduct a healthcare survey”.

Our records show that you were a recent ICU patient at (hospital). We are conducting a survey with family members or friends of recent ICU patients. Many patients are not very aware during their ICU experience, that is why we ask for feedback from the family member or friend who was most involved in your ICU experience. This information will help the ICU improve its service to patients.

*If there is a contact listed with the patient’s record:* I have the name of (contact), is this person who I should talk to about your ICU experience?

*Yes, and in that household:* May I speak with them? *If not available, schedule a callback.*

*Yes, but NOT in that household:* Thank you, I will call them. Is this their correct phone number as far as you know *(read phone number and correct if needed)*? Thank you for your time. If you spent time in another area of the hospital after your ICU experience, you may receive a phone call asking you to complete a survey about that nursing area. Have a good (day/evening).

*No, but in that household:* May I speak with them? *If not available, schedule a callback.*

*No, and NOT in that household:* Can you tell me the name and phone number of the person we can talk to about your ICU experience? Thank you for that information, I will give them a call. If you spent time in another area of the hospital after your ICU experience, you may receive a phone call asking you to complete a survey about that nursing area. Have a good (day/evening).

*If there is NOT a contact listed with the patient’s record:* Can you tell me the name of the person we can talk to about your ICU experience?

*Yes, and in that household:* May I speak with that person? *If not available, schedule a callback.*

*Yes, but not in that household:* And can I get their phone number? Thank you for that information, I will give them a call. If you spent time in another area of the hospital after your ICU experience, you may receive a phone call asking you to complete a survey about that nursing area. Have a good (day/evening).

*If patient would like to complete the survey:* Do you recall your entire experience in the ICU well enough to complete the survey? And can you separate your ICU experience from your other areas of the hospital you may have spent time in? *If patient feels they meet the criteria, proceed to START OF SURVEY. If patient does not recall well enough, then ask:* Would it be alright if we talked to the family member or friend who was most involved in your ICU experience? *If yes, go to appropriate screen depending upon whether you have contact information. If no, then terminate:* Thank you for your time. If you spent time in another area of the hospital after your ICU experience, you may receive a phone call asking you to complete a survey about that nursing area. Have a good (day/evening).

***ADULT FAMILY MEMBER OR FRIEND INTRODUCTION:***

Hello, this is ________ calling on behalf of (hospital). May I speak with (family member or friend)? *If asked for purpose of call:* “We are calling to conduct a healthcare survey”.

We are conducting a survey with family members or friends of recent ICU patients and your name was given to us by (patient). We are interested in your opinions about the care (patient name) received in the ICU at (hospital). Many patients are not very aware during their ICU experience, that is why we ask for feedback from the family member or friend who was most involved in the patient’s ICU experience. This information will help the ICU improve its service to patients. Would you help us by answering a few questions?

***START OF SURVEY:***

I will ask you to rate some aspects of (your/your patient’s) ICU experience in terms of whether they were *Excellent, Very Good, Good, Fair,* or *Poor* (*Interviewer Note: Please pause momentarily between the ratings when reading them to the respondent.*) You may also indicate if you have no opinion or if the statement does not apply to your situation. Of course, your answers will be completely confidential.

Although (you/your patient) may have spent time in another nursing unit while in the hospital, please answer the questions thinking only about (your/your patient’s) experience in the (ICU name).

These first few questions are about the care (you/your patient) received from the nurses in the ICU. How would you rate...

**Very DK/**

**Poor Fair Good Good Excellent NA**

1. The genuine caring and concern shown to you (and your patient) by the 1 2 3 4 5 6

ICU nurses. Would you say that it was: *Excellent, Very Good, Good,*

*Fair*, or *Poor*?

1. How well the ICU nurses explained things to you. 1 2 3 4 5 6
2. The skills and knowledge of the ICU nurses. 1 2 3 4 5 6
3. How well the ICU nurses listened and seriously considered what you were 1 2 3 4 5 6

saying to them.

1. How well the ICU nurses followed through when asked to do something. 1 2 3 4 5 6

The next questions are about the care (you/your patient) received from the physician who spent the most time with (you/your patient) during (your/their) ICU stay. Again, thinking only about the ICU, can you tell me the name of the physician who spent the most time with (you/your patient) during (your/their) ICU stay? *Select name from the list. If “other”, please specify. If “unsure”, read the list of names.*

Now, thinking about that physician, how would you rate...

1. The genuine caring and concern shown to you (and your patient) by that physician. 1 2 3 4 5 6
2. The skills and knowledge of that physician. 1 2 3 4 5 6
3. How well that physician explained things to you. 1 2 3 4 5 6
4. How well that physician listened and seriously considered what you were saying. 1 2 3 4 5 6

Next, how would you rate...

1. The caring and concern of the social workers or care managers. 1 2 3 4 5 6
2. The skills and knowledge of the social workers or care managers. 1 2 3 4 5 6
3. The caring and concern of the respiratory therapists. 1 2 3 4 5 6
4. The skills and knowledge of the respiratory therapists. 1 2 3 4 5 6
5. The helpfulness of the ICU unit clerks. 1 2 3 4 5 6

*This question asked only of patients at one hospital*

Now, I would like you to rate the care (you/your patient) received from all the ICU staff in general. How would you rate...

1. How well your (patient's) privacy was respected by all the ICU staff. 1 2 3 4 5 6
2. How well all the ICU staff worked together to coordinate different 1 2 3 4 5 6

aspects of your (patient's) care.

1. The efforts of the ICU staff to involve you in decisions about your 1 2 3 4 5 6

(patient’s) care

1. How well the staff prepared you (and your patient) to leave the ICU. 1 2 3 4 5 6
2. And how would you rate the cleanliness of your (patient’s) ICU room. 1 2 3 4 5 6
3. *If someone other the patient is completing the interview:* Did you ever spend the night in your patient’s ICU room?

1 Yes *Continue to Q21*

2 No *Skip to Q22*

**DK/ Never Sometimes Usually Always NA**

1. How often was the area around your (patient’s) ICU room quiet at night? 1 2 3 4 6

Would you say: *Never, Sometimes, Usually, or Always*?

The next few questions are about your (patient’s) medical care. How would you rate...

**Very DK/**

**Poor Fair Good Good Excellent NA**

1. How well your (patient’s) pain was controlled while in the ICU. 1 2 3 4 5 6

Would you say that it was: *Excellent, Very Good, Good, Fair*, or *Poor*?

**Not at All Extremely DK/**

**Confident Confident NA**

1. How confident were you that the ICU was providing the very best MEDICAL 1 2 3 4 5 6

care possible? Please answer on a scale of one to five where one means

*Not at all Confident* and five means *Extremely Confident*.

**Very DK/**

**Poor Fair Good Good Excellent NA**

1. Now, please rate the OVERALL quality of care and services you (and your 1 2 3 4 5 6

patient) received while in the ICU. Would you say that it was:

*Excellent, Very Good, Good, Fair*, or *Poor*?

26. Did a manager or supervisor come into your room to visit with you for a few minutes?

1 Yes

2 No

6 DK./NA

WHO WAS INTERVIEWED?

1 Spouse

2 Parent

3 Other Family Member

4 Other

5 Patient

*Version ICU.Q6c*

©2013, Intermountain Healthcare Hospitals Inc. All rights reserved. Reproduction or translation of any part of this work beyond that permitted by the United States Copyright Act without permission of the copyright owner is unlawful. All permitted reproductions or translations must include the following copyright notice on each page: © 2013, Intermountain Healthcare Hospitals, Inc.
